# Elevated ACKR2 expression is a common feature of inflammatory arthropathies

**DOI:** 10.1093/rheumatology/kex176

**Published:** 2017-05-09

**Authors:** Helen M. Baldwin, Mark D. Singh, Veronica Codullo, Vicky King, Hilary Wilson, Iain McInnes, Gerard J. Graham

**Affiliations:** 1 Institute of Infection, Immunity and Inflammation, Glasgow Biomedical Research Centre, College of Medical, Veterinary and Life Sciences, University of Glasgow, Glasgow, UK

**Keywords:** chemokine, ACKR2, arthritis, inflammation

## Abstract

**Objectives:**

Chemokines are essential contributors to leucocyte accumulation at sites of inflammatory pathology. Interfering with chemokine or chemokine receptor function therefore represents a plausible therapeutic option. However, our currently limited understanding of chemokine orchestration of inflammatory responses means that such therapies have not yet been fully developed. We have a particular interest in the family of atypical chemokine receptors that fine-tune, or resolve, chemokine-driven responses. In particular we are interested in atypical chemokine receptor 2 (ACKR2), which is a scavenging receptor for inflammatory CC-chemokines and that therefore helps to resolve *in vivo* inflammatory responses. The objective of the current study was to examine ACKR2 expression in common arthropathies.

**Methods:**

ACKR2 expression was measured by a combination of qPCR and immuno-histochemistry. In addition, circulating cytokine and chemokine levels in patient plasma were assessed using multiplexing approaches.

**Results:**

Expression of ACKR2 was elevated on peripheral blood cells as well as on leucocytes and stromal cells in synovial tissue. Expression on peripheral blood leucocytes correlated with, and could be regulated by, circulating cytokines with particularly strong associations being seen with IL-6 and hepatocyte growth factor. In addition, expression within the synovium was coincident with aggregates of lymphocytes, potentially atopic follicles and sites of high inflammatory chemokine expression. Similarly increased levels of ACKR2 have been reported in psoriasis and SSc.

**Conclusion:**

Our data clearly show increased ACKR2 in a variety of arthropathies and taking into account our, and others’, previous data we now propose that elevated ACKR2 expression is a common feature of inflammatory pathologies.

Rheumatology key messages

- ACKR2 expression is elevated within peripheral blood mononuclear cells from patients with a PsA and RA.
- CKR2 expression correlates with, and can be induced by, inflammatory cytokines and CC-chemokines in RA.
- Elevated ACKR2 expression is a consistent feature of inflammatory disease.

## Introduction

The *in vivo* migration of leukocytes is regulated by proteins belonging to the chemokine family [1]. This family is defined on the basis of the presence of a conserved cysteine motif in the mature protein sequence and is further divided into CC, CXC, XC and CX3C subfamilies according to the specific nature of the cysteine motif. Chemokines can be broadly classified as being either inflammatory or homeostatic depending on the contexts in which they function [2, 3]. Inflammatory chemokines are predominantly involved in recruiting inflammatory leucocytes to damaged or infected tissue sites, whereas homeostatic chemokines are involved in more precise tissue localization of leucocytes. Chemokines interact with their target cells through receptors belonging to the seven transmembrane spanning family of G-protein-coupled receptors [4]. There are 19 identified chemokine receptors and, again, these are broadly distributed into inflammatory and homeostatic subclasses. Given the essential roles played by chemokines and their receptors in regulating immune and inflammatory leucocyte migration, it is not surprising that they play essential roles in a broad range of autoimmune and inflammatory pathologies [5, 6]. Disappointingly, despite two decades of study, there are currently no antagonists of chemokine receptor function licensed for use in inflammatory diseases. This represents a significant failing in the field and is likely to be related to our currently poorly developed understanding of the full complexities of the chemokine orchestration of inflammatory responses [6].

In addition to the classical signalling chemokine receptors there exists a separate subclass of receptors characterized by an inability to mount typical signalling responses to chemokine binding [7]. These receptors also possess an altered DRYLAIV motif in the second intracellular loop and have been named atypical chemokine receptors on this basis. There are currently four members of the atypical chemokine receptor family [7–9]: ACKR1 (formerly known as DARC), ACKR2 (formerly known as D6 or ccbp2), ACKR3 (formerly known as RDC1 or CXCR7) and ACKR4 (formerly known as CCRL1 or CCXCKR). These molecules serve essential *in vivo* functions in fine-tuning, or resolving, chemokine-driven responses and represent important new contributors to the overall regulation of chemokine function.

We have a particular interest in ACKR2, which is a highly promiscuous receptor for inflammatory CC-chemokines and which is expressed by lymphatic endothelial cells, syncytiotrophoblasts and some subsets of leucocytes [10, 11]. Following ligand binding, ACKR2 internalizes ligands and targets them for intracellular degradation [12, 13]. It is therefore a scavenging receptor for inflammatory CC-chemokines and its broad promiscuity ensures that it can scavenge essentially all CC-chemokines involved in inflammatory responses. Work from us, and others, has demonstrated a clear role for ACKR2 in the resolution of inflammatory responses at all tissue sites at which it is expressed [14–20]. In addition we recently demonstrated a role for ACKR2 in lymphatic vessel development [21]. We have also previously shown marked up-regulation of ACKR2 expression in the epidermis and peripheral blood in psoriasis [22], and whole skin and peripheral blood in systemic sclerosis [23]. Others have further shown ACKR2 overexpression in chronic obstructive pulmonary disease [24] and post-myocardial infarction tissues [25] suggesting an, as yet poorly defined, association with inflammatory disease. The purpose of the current study was to extend these analyses to incorporate inflammatory arthropathies, where ACKR2-binding inflammatory CC-chemokines such as CC chemokine ligand (CCL) 1, 2, 3 and 5 play key roles in the propagation and maintenance of synovitis [26–32].

## Methods

### Ethics statement

This study was approved by the West of Scotland Research Ethics Committee and the University of Glasgow Medical, Veterinary and Life Sciences Ethics Committee. Written consent was obtained from all participants according to the Declaration of Helsinki.

### Isolation of peripheral blood mononuclear cells and plasma

Heparinized whole blood (10 ml) was taken from healthy donors and inflammatory arthritis patients (early RA, established RA and PsA). Matched serum samples (10 ml) were taken from each patient. Heparinized blood was diluted 1:1 with wash buffer [PBS supplemented with 1% fetal calf serum (FCS) and 2 mM ethylenediaminetetraacetic acid (EDTA)] and layered onto Histopaque (cat no. 1077-1, Sigma-Aldrich, St Louis, MO, USA) according to the manufacturer’s instructions. Blood was centrifuged at 400 *g* for 30 min (with the brake removed) and the buffy layer was extracted. Cells were washed twice with wash buffer and counted using a haemocytometer. Cells were either resuspended in RLT buffer (cat. no. 74004, Qiagen, Hilden, Germany), containing β-mercaptoethanol, and stored at −80°C for future RNA isolation or resuspended at 1 × 10^6^/ml in complete media (RPMI + 10% FCS + 2 mM l-glutamine + penicillin/streptomycin) for future cell culture. Plasma samples were centrifuged at 500g for 10 min at 4°C and serum was stored in 500 μl aliquots at −80°C for later analysis.

### Quantitative real time RT-PCR

Peripheral blood mononuclear cell (PBMC) samples stored in RLT buffer at –80°C were thawed at room temperature and RNA was extracted using the RNeasy micro kit (Qiagen cat. no. 74004) according to the manufacturer’s instructions. RNA concentrations and quality were measured using the Nanodrop-2000 (Thermo Fisher Scientific, Waltham, MA, USA) and stored at −80°C for future cDNA synthesis. RNA was thawed and was reverse-transcribed to cDNA (500 ng) using Affinity Script (cat. no. 600559, Agilent Technologies, Santa Clara, CA, USA) according to the manufacturer’s instructions. cDNA was stored at −20 °C until used for quantitative real-time RT-PCR (qPCR) using methodology and primers as described previously [33–35]. Samples were run for 40 cycles on the 7900HT Fast Real-Time PCR System (ABI) and data were analysed using ABI Prism SDS software (Thermo Fisher Scientific). The absolute number of ACKR2 copies was normalized to TATA-binding protein copy number. 

### Quantification of cytokines and chemokines

Plasma cytokines and chemokines were quantified using the human cytokine 30-plex luminex kit (cat. no. LHC6003, Thermo Fisher Scientific). The serum was analysed undiluted. The detection level of the luminex was 30 pg/ml; all readings lower than this were designated non-detectable.

### Immunofluorescence staining of synovial tissue

Paraffin-embedded RA synovial tissue was sliced into 5 μm sections and mounted onto Superfrost slides (cat. no. 631-0108 VWR, Hunter Boulevard, Magna Park, Lutterworth, Leicestershire, UK). Sections were rehydrated to water through xylene and successive concentrations of alcohol. Antigen was retrieved by boiling slides in 0.05 M citrate buffer (pH 6) for 8 min before slides were blocked for 30 min at room temperature (RT) in 20% horse serum (cat. no. S-2000, Vector Laboratories, Burlingame, CA, USA) and biotin block (Vector Laboratories cat. no. SP-2001) diluted in Tris-buffered saline containing 0.01% Tween-20 (TBST). Slides were then incubated overnight at 4°C with rabbit anti-human CCBP2 (ACKR2, 2.5 μg/ml, Sigma-Aldrich cat. no. HPA013819) at 4°C in Dako REAL antibody diluent (cat. no. S2022, Dako, Carpenteria, CA, USA) supplemented with 2.5% horse serum–2.5% human serum (Sigma-Aldrich cat. no. H4522) and biotin block (Vector Laboratories cat. no. SP-2001). The following morning, slides were washed in TBST and incubated for a further 30 min at RT with biotinylated horse anti-rabbit IgG (1/200, Vector Laboratories cat. no. BA-1100) in Dako REAL antibody diluent supplemented with 2.5% horse serum–2.5% human serum. Slides were washed in TBST and incubated with Fluorescein Avidin-D (1/500, Vector Laboratories cat. no. A2001) for 40 min at RT.

Slides were then washed twice in TBST, and re-blocked with 20% horse serum (Vector Laboratories cat. no. S-2000) and Avidin-D block (Vector Laboratories cat. no. SP-2001) diluted in TBST for 30 min at RT. Slides were washed and stained with antibodies against lineage markers overnight at 4°C in order to detect ACKR2^+^ macrophages: mouse IgG1 anti-human CD68 (clone PG-M1, 1 µg/ml; Dako cat. no. M0876); mast cells: mouse IgG anti-human mast cell tryptase (clone AA1, 0.43 µg/ml; Dako cat. no. M7052); CD3^+^ T cells: mouse IgG_1_ anti-human CD3 (clone LN10, 1 μg/ml, Vector labs cat. no. VP-C429); CD20^+^ B cells: mouse IgG_2a_ anti-human CD20cy (clone L26, Dako cat. no. M0755) or mouse IgG_1_ anti-human CCL3 (clone 93321, cat. no. MAB270, R&D Systems, Minneapolis, MN, USA). The following day, sections were incubated with biotinylated horse anti-mouse IgG H + L (Vector Laboratories cat. no. BA-2000) and subsequently stained with Avidin-D Texas Red (Vector Laboratories cat. no. A2006) for 40 min. Slides were washed, mounted using Vectashield mounting medium with 4′,6-diamidino-2-phenylindole (DAPI; Vector Laboratories cat. no. H1200) and analysed on an epifluorescence imaging microscope (Carl Zeiss, Oberkochen, Germany). Images were captured using Zeiss AxioVision software v. 4.8.2.

### Immunofluorescence staining of cell cytospins

PBMCs were isolated as described above and resuspended in PBS containing 2 mM EDTA at 0.5 × 10^6^/ml. Cells (0.1 × 10^6^) were spun onto superfrost slides (VWR) at 800 r.p.m. for 3 min using a Shadon Cytospin Centrifuge (Thermo Fisher Scientific), fixed in 100% methanol for 10 min at RT and left to air dry. Slides were then blocked with 20% horse serum (Vector Laboratories cat. no. S-2000) diluted in TBST, before being stained overnight at 4°C with rabbit anti-human CCBP2 (ACKR2, 2.5 μg/ml, Sigma-Aldrich cat. no. HPA013819), mouse IgG_1_ anti-human CD3 (Dako) and mouse IgG_2a_ anti-human CD20 (Dako). Antibodies were diluted in TBST supplemented with 2.5% horse serum (Vector Laboratories cat. no. S-2000) and 2.5% human serum (Sigma-Aldrich cat. no. H4522). The following day, slides were washed and incubated with goat anti-rabbit fluorescein isothiocyanate (1/300, cat. no. 4030-02), goat anti-mouse IgG_1_ Cy5 (1/300 cat. no. 1070-15) and goat anti-mouse IgG_2a_ tetramethylrhodamine (1/300, cat. no. 1080-03, all from Southern Biotech, Birmingham, AL, USA) in TBST containing 2.5% horse serum–2.5% human serum for 30 min at RT. Slides were washed, mounted using Vectashield mounting medium with DAPI (Vector Laboratories cat. no. H1200) and analysed on an epifluorescence imaging microscope (Carl Zeiss). Images were captured using AxioVision software v. 4.8.2.

### Stimulation of PBMCs to detect ACKR2 expression

PBMCs were cultured at 1 × 10^6^/ml in six-well plates in complete media (RPMI + 10% FCS+ 2 mM l-glutamine + penicillin/streptomycin) at 37°C for 6, 24 or 48 h with combinations of cytokines or chemokines, all from Peprotech (Rocky Hill, NJ, USA) at 100 ng/ml. Cells were harvested on ice, and processed for qPCR as described above.

### Statistical analysis

For all statistical tests, non-parametric data were analysed using the Mann–Whitney U test and parametric data using Student’s unpaired *t* test. To detect significant correlation between variables, Spearman’s correlation coefficient was used, where r = 1 denotes a perfect positive correlation and r = −1 a perfect negative correlation. P < 0.05 denotes significant differences.

## Results

### Patient cohort

A summary of the patient details is shown in supplementary Table S1, available at *Rheumatology* Online. Patients classified as early RA (early inflammatory disease) were defined as having a disease course of < 1 year and had been taking DMARDs for <6 months. All patients with RA and PsA had been treated with DMARDs for the majority of the duration of their disease (over 1 year). There were no significant differences in ESR or CRP between the patient groups (supplementary Fig. S1A and B, available at *Rheumatology* Online).

### ACKR2 expression is elevated in PBMCs from inflammatory arthritis patients

**Fig. 1:**
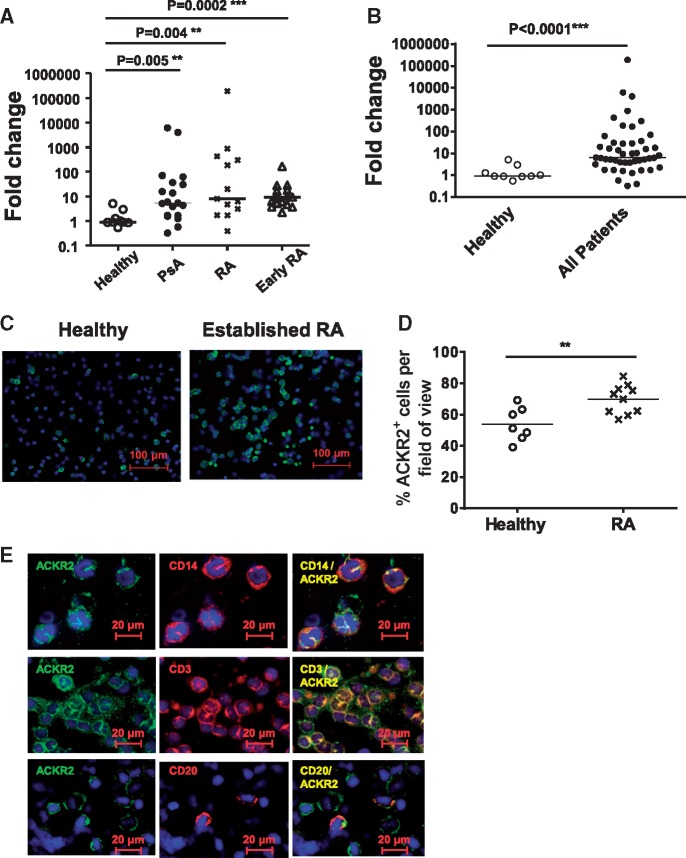
ACKR2 expression is elevated in peripheral blood mononuclear cells from inflammatory arthropathy patients (**A**) Atypical chemokine receptor 2 (ACKR2) expression in peripheral blood mononuclear cell (PBMCs) from PsA, RA and early RA patients compared with healthy controls. Data are expressed as fold change in expression relative to expression in healthy control samples, the mean value for which is set as 1. (**B**) Compiled data showing overall spread of ACKR2 expression across healthy controls and the three patient groups. Data are expressed as fold change in expression relative to expression in healthy control samples, the mean value for which is set as 1. (**C**) Cytospins of PBMCs from healthy controls and RA patients stained for ACKR2. (**D**) ACKR2^+^ cells per view over 10 fields of view. Mean expression is shown from seven healthy and 10 RA donors. (**E**) Co-staining for ACKR2 (green), and CD14, CD3 or CD20 (each in red) of RA patient PBMCs. The overlay is shown in the third panel of the images.

To determine the expression levels of ACKR2 in PBMCs from established PsA, RA or early RA in comparison with healthy controls, we used qPCR as previously described [33, 34]. As shown in Fig. 1A all three patient populations displayed significantly elevated PBMC ACKR2 expression relative to healthy controls. Specifically we found ACKR2 expression to be increased by a median of ∼2-fold in established PsA (P = 0.005), 5-fold in established RA (P = 0.004) and 9-fold in early RA (P = 0.0002). There was also a significant difference in ACKR2 expression between PsA and early RA patients (P = 0.04). Some patients displayed extremely elevated levels of ACKR2 expression and, overall, there was an almost five log spread in ACKR2 levels when data from healthy controls and all three arthropathy groups were pooled (Fig. 1B).

To attempt to identify the peripheral blood leucocyte subpopulations responsible for the elevated ACKR2 expression we performed immunostaining of PBMC cytospins from RA patients (Fig. 1C). Quantification of the numbers of ACKR2-positive cells per field of view revealed a highly significant increase in the percentage ACKR2-positive cells in RA patients compared with healthy controls (Fig. 1D). Co-staining for ACKR2 and CD14, CD3 or CD20 indicated that ACKR2 expression was detectable in peripheral blood monocytes, T cells and B cells (Fig. 1E). In contrast, while a few isolated cells with a neutrophilic morphology were seen to be positive for ACKR2 staining in healthy controls (supplementary Fig. S2A, available at *Rheumatology* Online), the majority of ACKR2-positive cells in the RA patients lacked this morphology (supplementary Fig. S2B, available at *Rheumatology* Online) suggesting little involvement of neutrophils in contributing to the elevated ACKR2 expression. Thus elevated PBMC ACKR2 expression is seen in a variety of leucocyte subtypes in a range of inflammatory arthropathies.

### ACKR2 expression levels do not correlate with age or markers of disease severity

While there were significant differences between the ages of the healthy controls and the patient groups, and also between patient groups (supplementary Fig. S1C, available at *Rheumatology* Online), there was no correlation between age and ACKR2 expression in PBMCs (supplementary Fig. S3A, available at *Rheumatology* Online). This suggests that age was not a contributing factor to the data obtained. We next tested for correlations between ACKR2 expression and acute phase response markers (ESR and CRP). This analysis showed no significant correlation between ACKR2 and ESR or CRP (supplementary Fig. S3B and C, available at *Rheumatology* Online). Thus PBMC ACKR2 expression levels do not correlate with age or disease severity in arthropathies.

### ACKR2 expression correlates with peripheral blood cytokine and chemokine levels

**Fig. 2:**
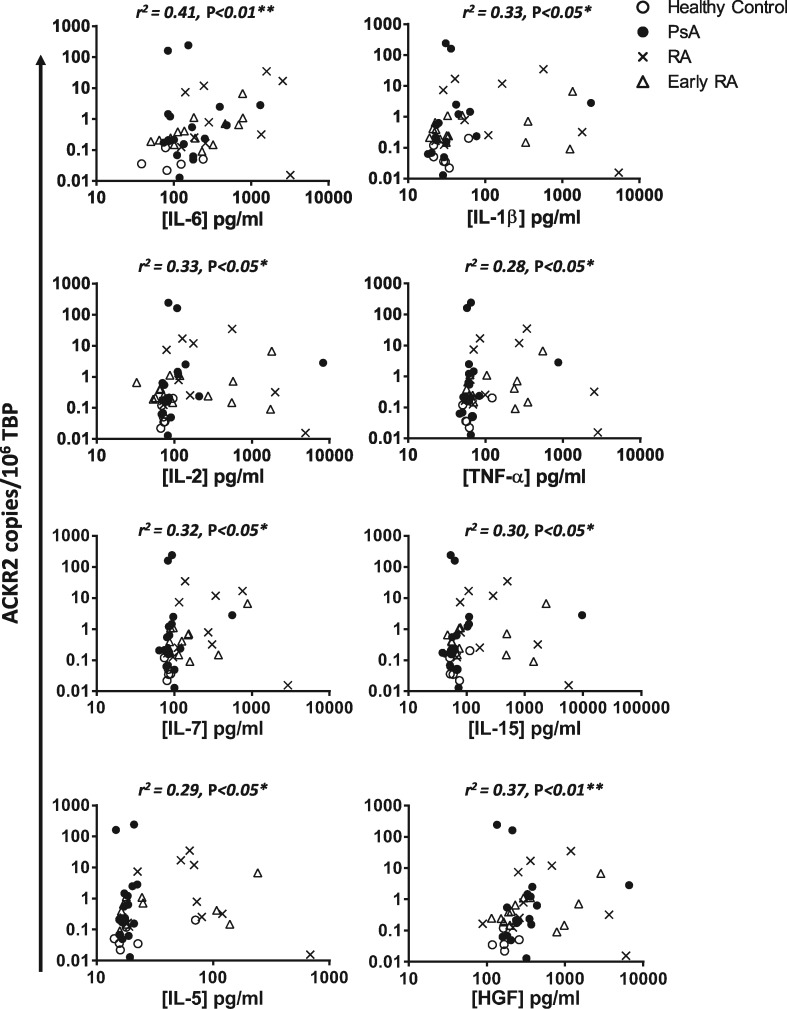
Correlation of ACKR2 expression in peripheral blood mononuclear cells with plasma cytokines Peripheral blood mononuclear cells (PBMCs) and paired serum samples were taken from healthy controls, PsA patients, RA patients or early RA patients. Atypical chemokine receptor 2 (ACKR2) expression was measured using qPCR with absolute quantification and normalized to 10^6^ copies of TATA-binding protein (TBP). Plasma was analysed by 30-plex luminex analysis. ACKR2 expression is shown against significantly correlating cytokines: IL-6, IL-1β, IL-2, TNF-α, IL-7, IL-15, IL-5 and hepatocyte growth factor (HGF). Correlation analysis was performed using Spearman’s correlation coefficient.

**Fig. 3:**
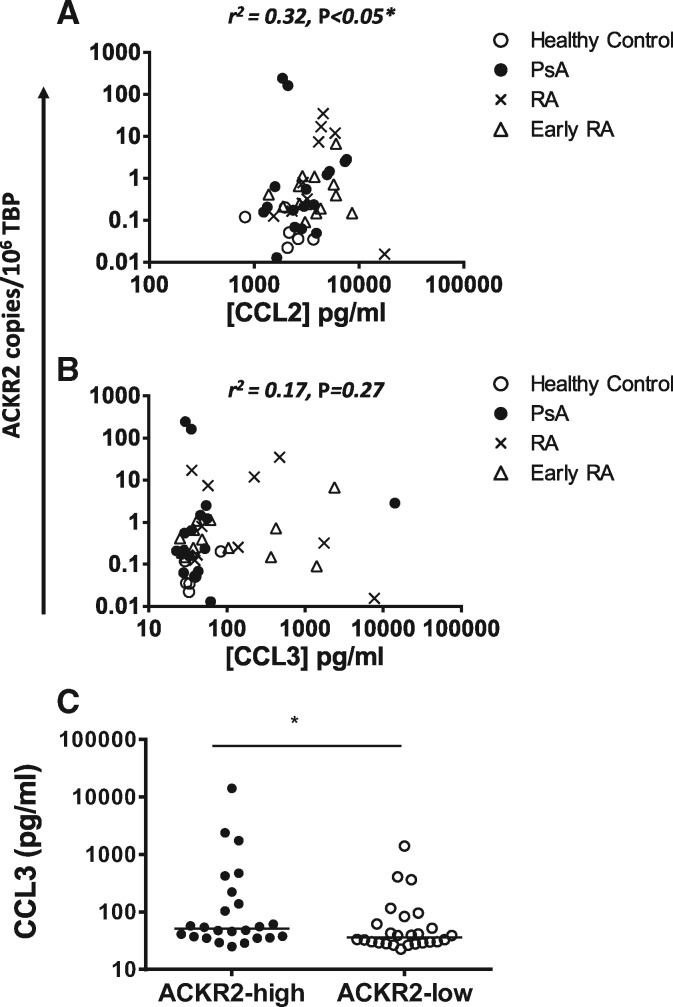
Correlation of ACKR2 expression in peripheral blood mononuclear cells with ACKR2-binding chemokines Peripheral blood mononuclear cells (PBMCs) and paired plasma samples were taken from healthy controls, PsA patients, RA patients or early RA patients. Atypical chemokine receptor 2 (ACKR2) expression was measured using qPCR with absolute quantification and normalized to 10^6^ copies of TATA-binding protein (TBP). Plasma was analysed by 30-plex Luminex analysis. **(A**, **B**) ACKR2 expression is shown correlated with CC chemokine ligand (CCL) 2 (**A**) and CCL3 (**B**). (**C**) CCL3 levels between ACKR2-high and ACKR2-low patients. Correlation analysis was performed using Spearman’s correlation coefficient.

To attempt to define possible regulators of ACKR2 expression in PBMCs, we measured levels of a broad range of circulating cytokines and chemokines using Luminex and tested for correlations with PBMC ACKR2 expression levels. As shown in Fig. 2, when data from all patient and control groups were combined, statistically significant correlations with ACKR2 expression levels were seen for IL-6, IL-1β, IL-2, TNF, IL-7, IL-15, IL-5 and hepatocyte growth factor (HGF). Each correlation detected was positive in the sense that increased cytokine levels correlated with increased ACKR2 levels. By far the most significant correlations were with IL-6 and HGF concentrations. In addition, for chemokines, there was a positive correlation between CCL2 levels and ACKR2 expression (Fig. 3A) and while no overall correlation was detected with CCL3 (Fig. 3B), patients expressing ACKR2 levels above the median of the whole population display significantly higher CCL3 levels than those below the median (Fig. 3C). Interestingly, when broken down to disease subtypes, the strongest correlation between IL-6 levels and ACKR2 expression was seen for early RA and between IL-1 and IL-2 and ACKR2 for PsA.

Thus ACKR2 expression in PBMCs correlates positively with circulating levels of select cytokines and chemokines.

### Combinations of cytokines and chemokines can upregulate ACKR2 expression in PBMCs

**Fig. 4:**
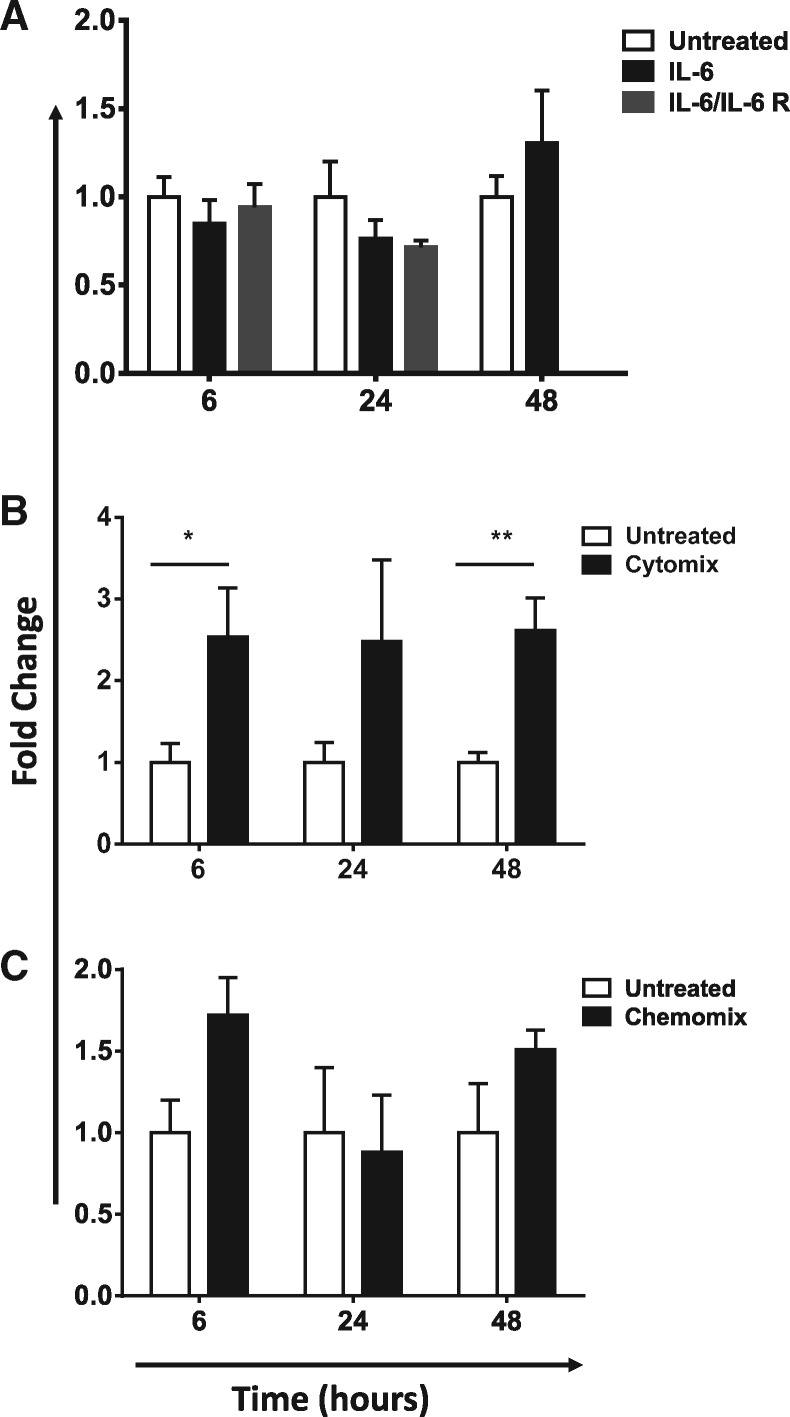
Regulation of ACKR2 expression by cytokines and chemokines (**A**) Healthy peripheral blood mononuclear cells (PBMCs) were treated with PBS or IL-6 in the presence or absence of soluble IL-6 receptor (IL-6R) for 6, 24 and 48 h. Atypical chemokine receptor 2 (ACKR2) expression assessed using qPCR. Data are expressed as ACKR2 copies/10^6^ copies of TATA binding protein. PBMCs were obtained from one donor. These data are representative of two replicate experiments. (**B**) Cytokines were pooled into a cytomix containing IL-6, IL-1β, IL-2, TNF-α, IL-7, IL-15, IL-5 and HGF (all at 100 ng/ml) and the mix used to stimulate PBMCs for 6, 24 and 48 h. Expression of ACKR2 was assessed as above. PBMCs were obtained from one donor. These data are representative of two replicate experiments. (**C**) A chemomix containing CC chemokine ligand (CCL) 2 and CCL3 (both at 10 μg/ml) was used to stimulate PBMCs, and ACKR2 expression measured. PBMCs were obtained from one donor. These data are representative of two replicate experiments.

To determine whether the correlating cytokines described above were functionally involved in regulating ACKR2 expression we took PBMCs from healthy individuals, with low levels of ACKR2, and tested the ability of cytokines to increase expression levels. As shown in Fig. 2, plasma IL-6 and HGF levels strongly correlated with PBMC ACKR2 levels. However as shown in Fig. 4A, IL-6 was unable to directly modulate ACKR2 expression levels in PBMCs. In a similar manner HGF was also unable to modulate expression levels (data not shown). Thus these cytokines are unable to independently induce ACKR2 expression over a 48 h time frame. Next, to test combinatorial cytokine regulation of ACKR2 expression, we combined the cytokines that correlated with ACKR2 to produce a cytomix containing IL-6, IL-1β, IL-2, TNF-α, IL-7, IL-15, IL-5 and HGF. In addition we generated a chemomix containing the chemokines CCL2 and CCL3. Healthy PBMCs were stimulated *in vitro* with these agents following which we detected a modest but significant and sustained increase in ACKR2 transcript levels after stimulation with the cytomix (Fig. 4B). To determine what contribution, if any, antigen activated T cells made to this increase in expression, we evaluated the impact of CD3 activation on ACKR2 expression by T cells. As shown in supplementary Fig. S4, available at *Rheumatology* Online, CD3 activation initially induced a modest decrease in expression that became significant by 24 h, but which was reversed at later time points when expression in resting and activating T cells was seen to be indistinguishable. In contrast to the cytomix, while the data presented in Fig. 4C reveal some modest significant differences in response to the chemomix, these were not consistent on a temporal basis and were not seen on repeat experiments. Overall therefore, we conclude that there is no increase in ACKR2 expression in PBMCs after stimulation with the chemomix. Thus PBMC expression of ACKR2 correlates with select circulating cytokines, and mixtures of these cytokines are capable of increasing ACKR2 expression, suggesting a functional association in peripheral blood.

### Expression of ACKR2 within RA synovium

**Fig. 5:**
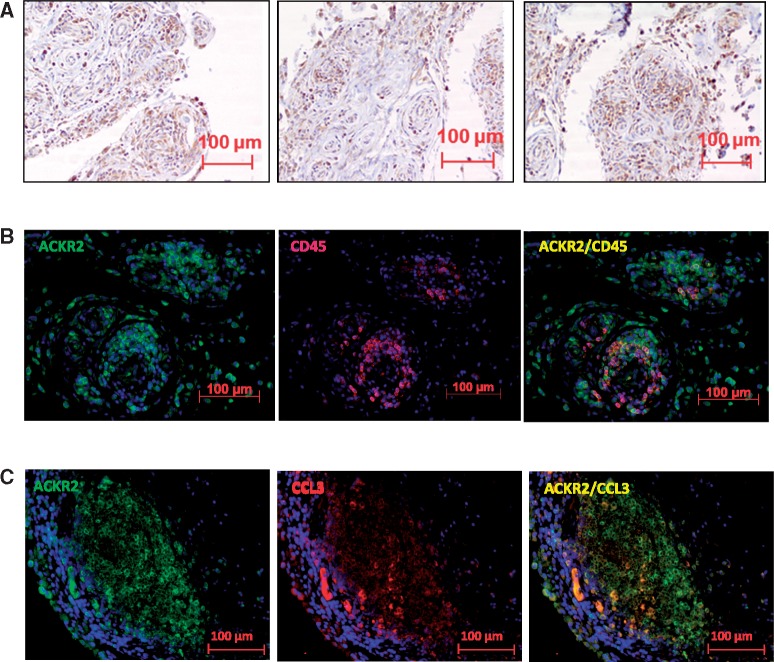
ACKR2 is expressed within the RA synovium in leucocytes and stromal cells (**A**) Paraffin embedded RA synovial tissue sections (5 μm) were stained for atypical chemokine receptor 2 (ACKR2). Slides were counterstained with haematoxylin. (**B**) Co-staining for ACKR2 (green) and CD45 (red) and showing the overlay of the two stains (yellow) on paraffin-embedded synovial tissue sections. Slides were counterstained with 4′,6-diamidino-2-phenylindole (DAPI; blue). (**C**) Paraffin embedded RA synovial tissue sections were stained for ACKR2 (green) and CC chemokine ligand 3 (CCL3; red). The third image shows the overlay of the two colours. Slides were counterstained with DAPI.

**Fig. 6:**
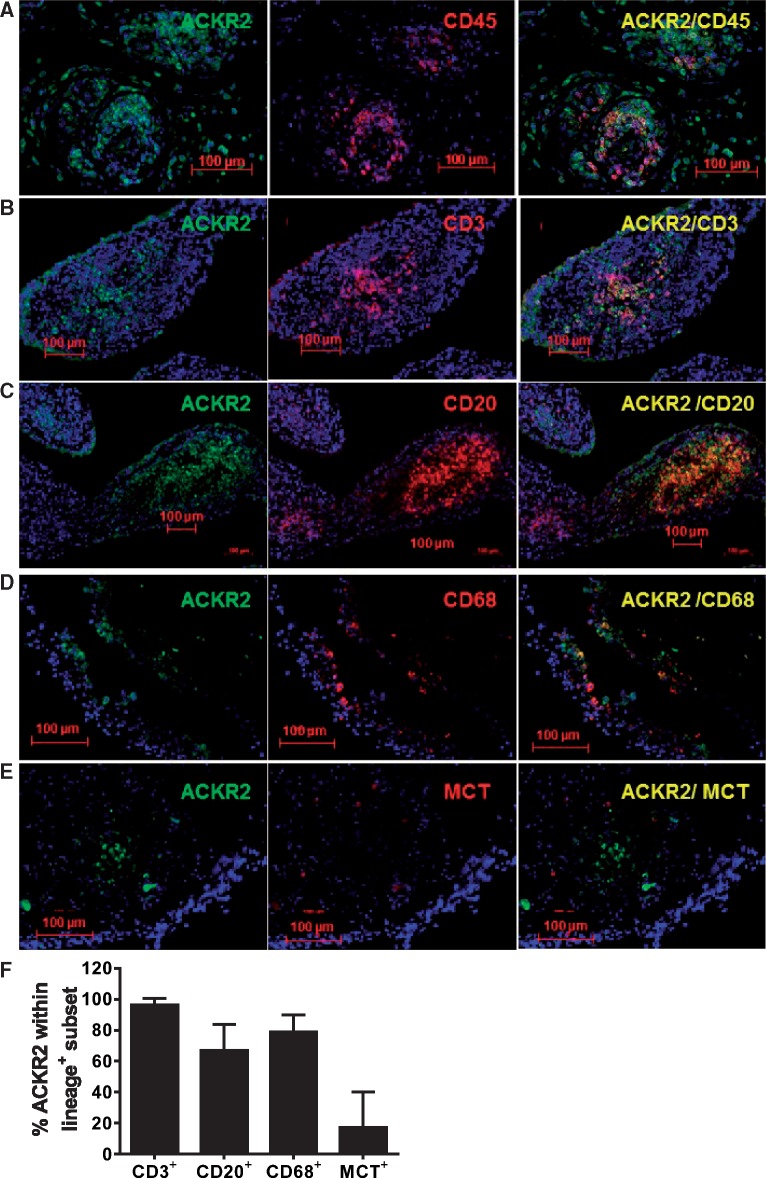
ACKR2 is expressed within RA synovium on defined leucocyte subpopulations (**A–E**) Paraffin embedded rheumatoid arthritis synovial tissue sections (5 μm) were stained for atypical chemokine receptor 2 (ACKR2) (green) together with CD45 (**A**), CD3 (**B**), CD20 (**C**), CD68 (**D**) or mast cell tryptase (MCT) (**E**) to identify leucocytes within the synovium expressing ACKR2. Yellow represents the overlay of the two colours and indicates coincident expression. Slides were counterstained with 4′,6-diamidino-2-phenylindole (DAPI) and visualized on a Zeiss epifluorescence microscope with AxioVision v. 4.8.2 software. (**F**) Percentages of ACKR2^+^ lineage^+^ cells quantified over 10 fields of view.

Next we used immunohistochemistry to examine expression of ACKR2 in RA synovium. Initial staining (Fig. 5A) revealed ACKR2-positive signals associated with cells at the tissue–synovial fluid interface as well as on numerous cells internal to the synovial tissue structure. Co-staining for CD45 and ACKR2 (Fig. 5B) demonstrated that the receptor is expressed by numerous leucocytes but also by stromal cells throughout the tissue. Interestingly, ACKR2-positive leucocytes were generally not randomly distributed within the tissue and were present predominantly within leucocyte aggregates. Notably, co-staining for ACKR2 and one of its ligands, CCL3, indicates that there is significant overlap in their expression patterns and that ACKR2 is therefore expressed at appropriate tissue positions for effective intra-synovial chemokine scavenging (Fig. 5C). We have previously reported expression of ACKR2 by T cells, B cells, monocytic cells and mast cells [33]. In agreement with this, co-staining for CD45 and markers specific to these leucocyte populations demonstrated ACKR2 expression on these cellular populations (Fig. 6A–D). Particularly strong co-staining was seen with aggregates of T and B cells (Fig. 6B and C) suggestive of ACKR2 expression within ectopic follicles. Thus ACKR2 is expressed in both leucocyte, and non-leucocyte, populations within the synovium, coincident with expression of inflammatory CC-chemokines.

## Discussion

ACKR2 is an important regulator of inflammation and we have previously reported marked upregulation of expression in skin and peripheral blood leucocytes in psoriasis [22] and SSc [23], and others have shown increased expression in chronic obstructive pulmonary disease [24] and post-myocardial infarction tissues [25]. The purpose of the present study was to extend these observations and to examine expression of ACKR2 in arthropathies. Here we present data demonstrating upregulation of ACKR2 in PsA, RA and early RA. We conclude, on this basis, that elevated peripheral blood ACKR2 expression is a consistent feature of inflammatory pathologies. We further demonstrate an association between select circulating cytokines and ACKR2 expression and show that mixtures of these cytokines are capable of increasing ACKR2 expression levels in healthy PBMCs. Notably, the increase in ACKR2 expression in response to cytokine treatment was modest. The fact that the cytokines were used at a high concentration, relative to their likely *in vivo* concentrations, and that cytokines do not demonstrate bell-shaped dose–response curves, suggests that it is likely that this modest increase is the maximum that can be achieved with this cytokine mix. The relevance of this increase remains to be determined, especially given that the differences detected are at the transcriptional level and not the protein level. One further consideration is whether sufficient time was given in these experiments for effective induction of ACKR2 expression by the cytokine mix. Our previous studies have shown robust induction of ACKR2 by a variety of cytokines in a variety of cell types within a period of 24–48 h [22, 33, 34, 36]. We therefore do not anticipate that prolonged incubation with the cytokine mix beyond 48 h will lead to increased ACKR2 expression. The strongest association between circulating cytokines and ACKR2 expression was seen for IL-6 and HGF although neither cytokine alone was capable of inducing ACKR2 expression. Interestingly, while a similar association with HGF was seen in psoriatic patients, these patients displayed no association between IL-6 and ACKR2 (data not shown). This contrasts with the ACKR2-inducing effects of IL-6 seen on lymphatic endothelial cells [34]. Together our observations suggest alternative molecular drivers for enhanced ACKR2 expression in different inflammatory pathologies and cell types. Surprisingly in the arthropathy groups studied (as well as in psoriatic patients) there is no apparent correlation between peripheral blood ACKR2 expression and disease severity (supplementary Fig. S3, available at *Rheumatology* Online). As circulating inflammatory CC-chemokines will predominantly have derived from the original inflamed site, one intriguing possibility is that ACKR2 limits the activity of circulating chemokines and therefore potentially ameliorates the development of chemokine-associated comorbidities (e.g. atherosclerosis) in patients. This possibility, however, remains to be tested.

In addition we demonstrate expression of ACKR2 in both leukocytes and resident stromal populations in patient synovial biopsies. The demonstration of expression on stromal cells, especially those at the synovium–SF interface, extends observations made in psoriatic skin that demonstrated inducible expression on keratinocytes [22]. We hypothesize that stromal cell expression serves to reduce inflammatory chemokine bio-availability, or to limit its domain of influence, and this is supported by the coincident expression of ACKR2 and CCL3 in synovial tissues. Strikingly, many of the leukocytes that are positive for ACKR2 expression are B and T cells present in large leucocyte aggregates within the synovial tissues. We propose that these correspond to ectopic follicles although the role of ACKR2 within these structures is not immediately apparent. It may be that ACKR2 is important to ensure the relative absence of inflammatory leucocytes within these ectopic lymphoid follicles [37, 38].

In summary, therefore, we provide evidence demonstrating upregulation of ACKR2 in peripheral blood cells and in cells within the synovium. This, coupled with previous observations from psoriasis and SSc patients suggests that elevated ACKR2 levels are a consistent feature of many human inflammatory pathologies. Further work is required to determine the functional implications of elevated expression in these pathological contexts.

## Supplementary Material

Supplementary Table S1 and Figures
