# Supplementary material for: Elevated ACKR2 expression is a common feature of inflammatory arthropathies
Source: Rheumatology (Oxford). 2017 May 9;56(9):1607–17. doi: 10.1093/rheumatology/kex176 (PMC5850605; doi:10.1093/rheumatology/kex176)
Supplement: Supplementary Table S1 and Figures [file rhe-16-1288-file003_kex176.docx]

**SUPPLEMENTARY DATA**

**Supplementary Table S1. Patient characteristics**

| **Parameter** | **Healthy controls** | **PsA** | **RA** | **Early inflammatory arthritis** |
| --- | --- | --- | --- | --- |
| Age, years , mean (SD) | 44.4 (5.9) | 48.9 (10.6) | 60.8 (10.5) | 57.7 (10.1) |
| Sex, F:M | 7:4 | 17:2 | 10:5 | 14:1 |
| Disease duration, years, mean (SD) | N/A | 11.4 (6.5) | 19.1 (6.9) | 0.7 (0.3) |
| Treatment  None  DMARDs  Etanercept  Adalimumab  Rituximab | N/A | 6  8  1  3  1 | 7  5  0  2  1 | 0  12  0  3  0 |
| ESR, mean (SD) | N/A | 19.1 (16.1) | 25.5 (51.4) | 19.2 (12.8) |
| CRP, mean (SD) | N/A | 16.16 (18.2) | 26.5 (28.8) | 7 (9.1) |
| RhF  +  -  Unknown | N/A | 1  17  1 | 9  6 | 4  9  2 |
| ACPA  +  -  unknown | N/A | 0  18  1 | 4  11 | 2  11  2 |
| Total number | 11 | 19 | 15 | 15 |

**Supplementary Figure S1. ESR, CRP and age of the individuals used in this study**

Patients were recruited from Stobhill hospital and the Victoria Infirmary, Glasgow. ESR (A), CRP (B) and age (C) were plotted for each patient group.

**Supplementary Figure S2. Magnified images for Figure 1C showing ACKR2+ve cells with a neutrophilic morphology**

PBMC: peripheral blood mononuclear cells.

**Supplementary Figure S3. Correlation between ACKR2 expression in PBMC and age, ESR and CRP**

Peripheral blood mononuclear cells (PBMC) were taken from healthy controls, psoriatic arthritis patients (PsA), rheumatoid arthritis patients (RA) or early rheumatoid arthritis patients (early RA). ACKR2 expression was measured using qPCR with absolute quantification and normalised to 10^6^ copies of TATA binding protein (TATA-BP). ACKR2 expression is shown against age (A), ESR (B) and CRP (C). Correlation analysis was performed using Spearman’s Correlation Coefficient.

**Supplementary Figure S4. The effects of anti-CD3 treatment on ACKR2 expression by CD4+ T cells**
